# Book Notices

**Published:** 1879-09-20

**Authors:** 


### *BOOK NOTICES.*

*ATLAS OF SKIN DISEASES*, by LOUIS A. DUHRING, M.D., Prof. of skin diseases in the Hospital of the University of Pennsylvania; Physician to the Dispensary for skin diseases. Philadelphia ; Dermatologist to the Philadelphia Hospital, etc.—Part V—Lippincott & Co. ; illustrating *scabies*, *herpes zoster*, *tinea*, *sicosis* and *eczema*.

We feel that we cannot too highly commend the above work to the profession—to the practitioner not less than to the student of medicine. No department of medical science is so much neglected and so little understood as that of dermatology. The illustrations in the above work are so life-like, so admirably executed and so beautiful, that the reader cannot fail to be charmed, interested and instructed. Such a work must tend greatly to relieve dermatology of the dulness and intricacy usually attached to it by the student, and impart to it an impulse of interest and attraction hitherto unknown.

*A CLINICAL TREATISE ON THE DISEASES OF THE NERVOUS SYSTEM*, by M. ROSENTHAL, Professor of the Diseases of the Nervous System at Vienna; with a preface by Prof. Charcot. Translated from the author's revised and enlarged edition, by Putzel M.D., Physician to the Class of Nervous Diseases, Bellevue Hospital, etc.—N. Y., Wm. Wood & Co., 1879.

This is an elegant octavo of 270 pages. The obscurity and difficulties attending the study of nervous diseases calls for just such works as is here presented. It will doubtless be welcomed by the profession. It is admirably written, illustrated, and replete with instructive and interesting matter.

*GUIDE TO THE EXAMINATION OF URINE, WITH SPECIAL REFERENCE TO THE DISEASES OF THE URINARY APPARATUS*, by R. B. HOFFMAN, Prof. at the University of Gratz, and ULTZMAN, Docent at the University of Vienna. From the second edition; translated and edited by F. Forchheimer, M.D., Professor of Medical Chemistry at the Medical College of Ohio; with illustrations. 195 pages, $1.50 to $2 00.—Peter C. Thompson, publisher, 179 Vine street, Cincinnati.

This is a highly interesting and practical work.

*TRANSACTIONS OF THE THIRTY-FOURTH ANNUAL MEETING OF THE OHIO STATE MEDICAL SOCIETY HELD AT DAYTON, JUNE 3d, 4th & 5th*, 1879.

A neat little work of 200 pages in cloth. The address of welcome was made by Dr. J. M. Weaver; was followed by the annual address of the retiring president, Dr. B. B. Leonard; then follows a number of interesting papers. The writers are as follows: Roberts Bartholow, M.D.; I. H. Buckner, M.D.; S. F. Forbes, M.D.; D. N. Kinsman, M.D.; I. C. Reeve, M.D.; P. S. Conner, M.D.; H. I. Herrick, M.D.; Geo. E. Walton, M.D.; S. C. Ayers, M.D.; I. F. Baldwin, M.D.

*PHYSIOLOGY AND HISTOLOGY OF THE CEREBRAL CONVULSIONS, ALSO POISONS OF THE INTELLECT*, by CHAS. RICHET, A.M., P.H.D., former intern of the Hospital of Paris. Translated by Edward P. Fowler, M.D.—New York, Wm. Wood & Co., 27 Great Jones Street; 1879.

This translation of the valuable work of Chas. Richet upon the new and interesting subject of the *Physiology and Histology of Cerebral Convulsions* is timely and appropriate. The learned author, in a note to the translator, uses the following complimentary expression: "He has my f ll appreciation of the compliment and of the conscientious and scholarly manner in which his labor has been performed." The work is an octavo volume of 170 pages, illustrated and very neatly gotten up.

MATERIA MEDICA AND THERAPEUTICS (vegetable kingdom), by CHARLES D. F. PHILLIPS, M.D, F. R. C. S. E., lecturer on *Materia Medica*, Westminster Hospital, London.—Edited and adapted to the U. S. Pharmacopea, by Henry G. Piffard, A.M., M.D., Professor of Dermatology, University of the City of New York; Surgeon to Charity Hospital, etc.—322 pages; Wm. Wood & Co., New York.

"This work," said the author in his original preface, "aims at bringing together in a moderate compass a more extensive series of facts respecting the action of drugs, and especially a more enlarged view of what has been done in other countries than will be found in the ordinary text books." Its present editor, Prof. Piffard, has, in a very able manner, condensed the original so as to bring it within the limits of a single volume of the present series, and to render it more useful to the American practitioner. From a cursory glance at this work we regard it as eminently instructive and practical.

TRANSACTIONS OF THE STATE MEDICAL SOCIETY OF ARKANSAS, AT ITS FOURTH ANNUAL SESSION—1879—AT LITTLE ROCK. A work of 93 pages.

The address of welcome was made by Dr. Geo. C. Hartt, responded to by Dr. E. T. Dale. The volume is made up of a number of interesting reports and papers. We read with interest the reports of Dr. W. H. Hairkins, Chairman of Delegation to American Medical Association; of Dr. Lenthicum, of the Commission State Board of Health; and report of Commission on Vital Statistics. We note also as valuable the papers of Dr. Shebley on Pneumonia; Dr. Dibbrell's Surgical Cases, Amputations of Knee Joint; Suggestions on Preventive Medicine, by Dr. Bentley; a Case of Dissection, by Dr. Jennings, and a Case of Strangulated Hernia, by Dr. Dale.

TRANSACTIONS OF THE MEDICAL ASSOCIATION OF GEORGIA.—Thirteenth Annual Session, at Rome, April 16, 17, 18, 1879.

A neatly gotten up volume of 220 pages.

The officers and principal committees were published in our May number. The following interesting papers appear in the volume before us:

President's Address, by Jno. Thad. Johnson, M.D.

Annual Oration, by E. H. Richardson, Jr., M.D.

Quarantine. Its Sanitary and Political Aspect in Relation to the Spread of Epidemic Diseases, by J. C. LeHardy, M.D.

Yellow Fever. Its Origin and Relation to other Malarial Fevers, by J. G. Westmoreland, M.D.

The Yellow Fever Germ on Coast and Inland—Ship and Railroad Quarantine, by Henry F. Campbell, M.D.

Uterine Flexions, and the Stem-Treatment by the Soft-rubber Spring-stem Pessary, by Henry F. Campbell, M.D.

Tobacco Poisoning and its Effects upon the Eye-sight, by A. W. Calhoun, M.D.

The Toxic Effects of Bromide of Potassium, by Charles H. Hall, M D.

Morbid Reflex Excitability, by A. W. Griggs, M.D.

Phytolacca Decandra as a Remedial Agent in Mastitis, by Wm. F. Holt, M.D.

Traumatic Tetanus, by A. R. Taylor, M.D.

Pelvic Peritonitis resulting from the Use of Hodges' closed level Pessary, by H. V. Johnson, M.D.

Malarial Hematuria, by W. O'Daniel, M.D.

A Case of Placenta Prævia, by A. A. Smith, M.D.

Resection of the shaft of the Tibia, by F. R. Calhoun, M.D.

Report of the Section on Surgery for the Sixth Congressional District, by P. A. Wright, M.D.

TRANSACTIONS OF THE MEDICAL AND CHIRURGICAL FACULTY OF THE STATE OF MARYLAND.—Eighty-first Annual Session, held at Baltimore, April, 1879.

A neat volume of 196 pages, containing the minutes and a number of valuable papers.

The opening address was by Prof. H. N. Martin. The officers elect for the ensuing year are as follows:

President:—Dr. Samuel C. Chew.

Vice-Presidents:—Drs. H. P. C. Wilson; Jas. A. Steuart.

Recording Secretary:—Dr. W. G. Regester.

Assistant Secretary :—Dr. G. Lane Taneyhill.
Corresponding Secretary :—Dr. J. Edwin Michael.
Treasurer :—Dr. Judson Gilman.
Executive Committee :—Drs. P. C. Williams ; Jas. C. Thomas ; Christopher Jonnston ; L. McLane Tiffany ; Chas. G. W. Maggill.

VEGETARIANISM, THE RADICAL CURE FOR INTEMPERANCE, by HARRIET P. FOWLER.—New York, M. L. Holbrook & Co., 1879. A work of 79 pages.

This little work contains many hints and practical suggestions bearing upon the question of food and intemperance. It will well repay perusal.

*EPITOME OF SKIN DISEASES*, with Formulas for Students and Practitioners, by TILBURY FOX, M.D., F. R. C. P., Physician to the Department for Skin Diseases, in University College Hospital ; Author of various works on Skin Diseases, etc. ; and T. C. FOX, M. B., B. A. (Cantab) ; Physician to St. George's and St James' Dispensary—Second American edition, enlarged and revised by the Authors.—Philadelphia, Henry C. Lea, 1879.

*DISEASES OF THE INTESTINES AND PERITONEUM*, by JOHN LYER BRISTOW, M. D. , J. R. WARDELL, M. D. ; J. W. BIGBIE, M. D. ; S. O. HABERSHON, M. D. ; T. B. CURLING, F. R. S., and W. H. RANSOM, M. D., New York.—Wm. Wood & Co., 1879.

This is a neat work of 240 pages, containiug much useful and instructive matter in relation to the various important diseases of the intestines, peritoneum, etc. It is eminentely practical and should be inthedan of every progressive practitioner.

